# Supplementary material for: Chromosome-Level Genome Assembly Provides Insights into the Evolution of the Special Morphology and Behaviour of Lepturacanthus savala
Source: Genes (Basel). 2023 Jun 15;14(6):1268. doi: 10.3390/genes14061268 (PMC10298005; doi:10.3390/genes14061268)
Supplement: Supplementary file 1 [file genes-14-01268-s001.zip › genes-2419528-supplementary.pdf]

Supplementary Materials

Chromosome-level genome assembly provides insights into the evolution of the special morphology and behaviour of *Lepturacanthus savala*

Ren-Xie Wu*, Ben-Ben Miao, Fang-Yuan Han, Su-Fang Niu, Zhen-Bang Liang, Qing-Hua Wang and Yan-Shan Liang

College of Fisheries, Guangdong Ocean University, Zhanjiang 524088, China;
benben.miao@outlook.com (B.B.M.); hanfangyuan1999@163.com (F.Y.H.);
wolf0487@126.com(S.F.N); liangzhenbang0403@163.com (Z.B.L.);
wqh31016877430@163.com(Q.H.W.);yanshan-liang@outlook.com(Y.S.L.);
* Correspondence: wurenxie@163.com

Table S1. Summary statistics for contigs and scaffolds that at the sequence and chromosome levels.

Level	Type	Total length (bp)	Total number	N50 length (bp)	N50 number	N90 length (bp)	N90 number
Sequences	Contig	790,022,946	215	19,013,249	16	3,654,334	48
	Scaffold	790,022,946	215	19,013,249	16	3,654,334	48
Chromosomes	Contig	790,022,946	219	19,013,249	16	3,585,864	50
	Scaffold	790,034,746	101	32,774,443	11	23,501,047	22

Table S2. Types and counts of various Di-Tags identified during filtering

Di-Tag Type	Di-Tags Number
Total Di-Tags	6,124,460
Valid Di-Tags	5,365,963
Same circularized Di-Tags	10,484
Same fragment dangling ends Di-Tags	16,106
Same fragment internal Di-Tags	36,221
Re-ligation Di-Tags	147,118
Contiguous Di-Tags	97,223
Wrong size Di-Tags	451,345

Table S3. Overview of genome-wide repeat sequences

Repeat Type	RepeatMask length (bp)	Percent	ProteinMask length (bp)	Percent	Combined length (bp)	Percent	TRF length (bp)	Percent
Tandem							96,078,789	12.16%
DNA TE	119,984,228	15.19%	10,687,843	1.35%	123,793,417	15.67%		
LINE TE	41,633,842	5.27%	14,062,603	1.78%	47,584,557	6.02%		
SINE TE	18,805,604	2.38%	0	0%	18,805,604	2.38%		
LTR TE	101,322,579	12.83%	7,690,710	0.97%	102,555,646	12.98%		
Unknown	9,710,987	1.23%	0	0%	9,710,987	1.23%		
Total	271,664,381	34.39%	32,390,092	4.10%	277525905	35.13%		

Table S4. Annotation and detailed summary statistics of non-coding RNA

ncRNA Type		Copy	Average length (bp)	Total length (bp)	Percent (%)
miRNA		1,434	100.33	143,870	0.018211
tRNA		9,086	75.49	685,943	0.086824
rRNA	rRNA	10,263	204.63	2,100,089	0.27
	18S	534	843.33	450,338	0.057002
	28S	1,571	508.42	798,729	0.1
	5.8S	251	155.26	38,970	0.004933
	5S	7,907	102.7	812,052	0.1
snRNA	snRNA	1,328	147.45	195,807	0.024785
	CD-box	131	112.66	14,758	0.001868
	HACA-box	93	144.27	13,417	0.001698
	Splicing	1,090	151.22	164,829	0.020864

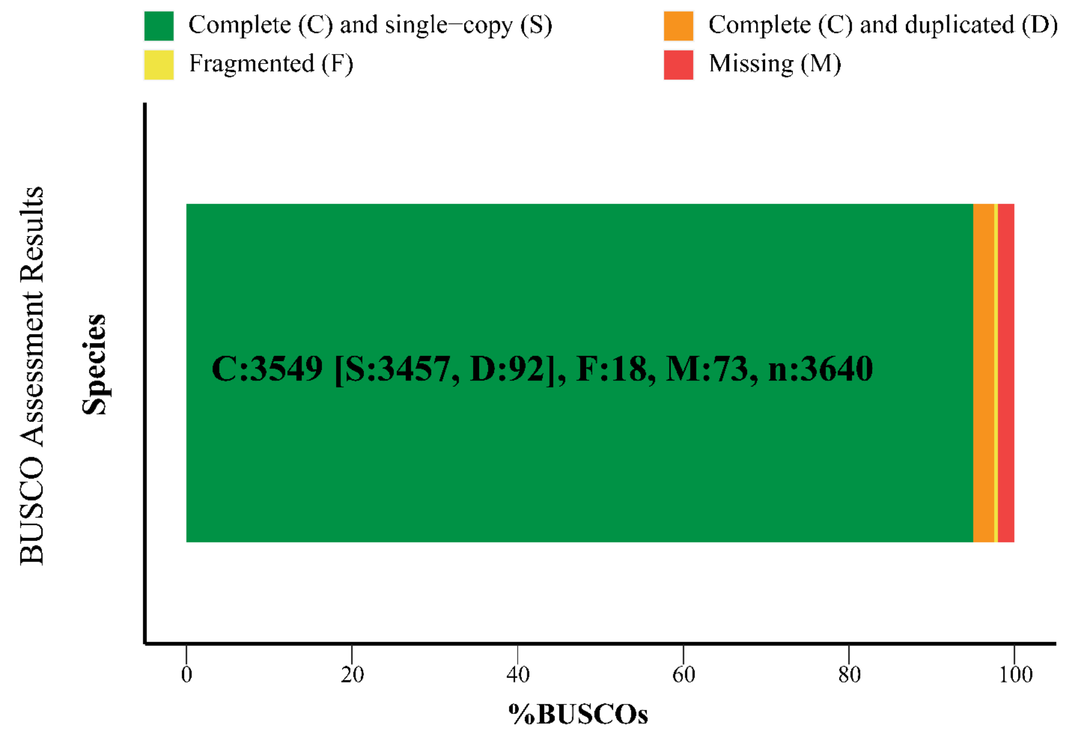

Figure S1. BUSCO genome integrity assessment and GC content distribution.

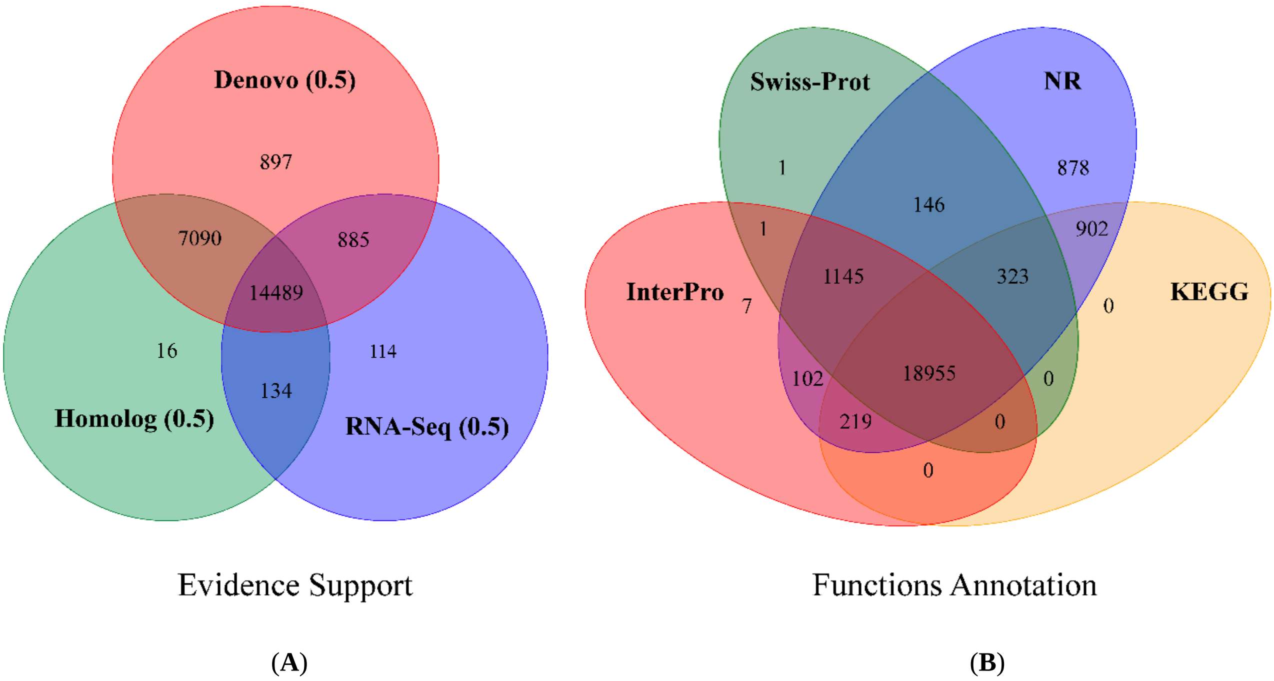

Figure S2. Genes identified by genome-wide de novo prediction, homologous prediction, and RNA sequencing data-based genes prediction (**A**). Functional annotation of genes based on NR, SwissProt, KEGG, InterPro databases (**B**).

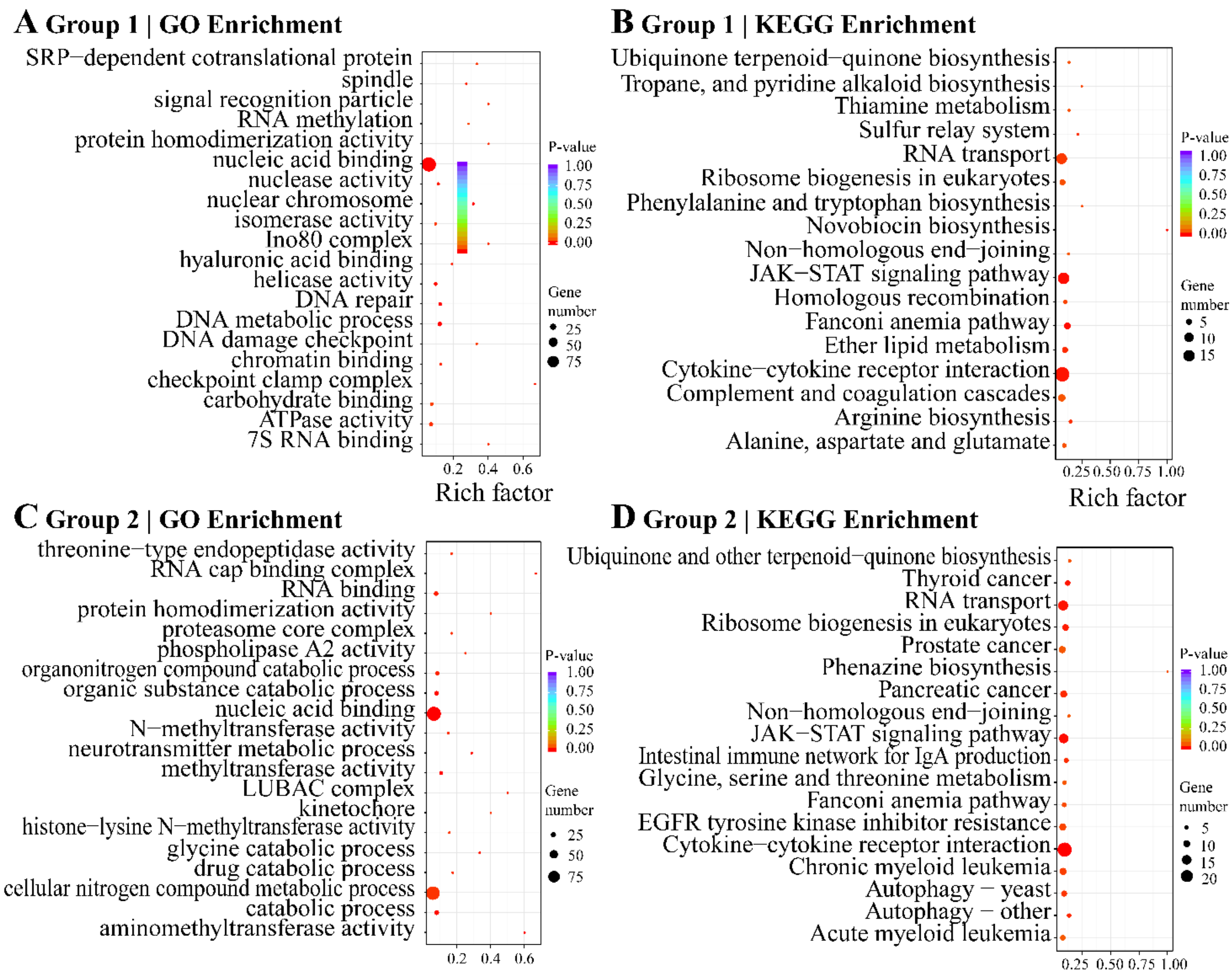

Figure S3. GO terms and KEGG pathways for genes screened in two groups of positive selection analyses.
